# DMS: Delusional Misidentification Syndrome or Dead Moneyman and Sex Offender? A Case Report of Reverse Capgras Syndrome

**DOI:** 10.1155/2022/9703482

**Published:** 2022-04-20

**Authors:** Elizabeth Kim, Rachael Murphy, Maggie Driscoll

**Affiliations:** Lehigh Valley Health Network, Bethlehem, PA, USA

## Abstract

Delusional misidentification syndromes (DMSs) are delusional phenomena where individuals believe that one has been altered or replaced. Here, we present the case of Ms. JS, who exemplifies one such DMS, Reverse Capgras Syndrome, which refers to the delusion that one has been replaced by an imposter. She endorsed psychosis and suicidality centered on her belief that she was in fact American financier and convicted sex offender Jeffrey Epstein. Her delusion was eventually resolved with medication management and therapy. In this report, we review Reverse Capgras Syndrome in the context of existing research on trauma-related pathology and the neural basis of self. We also demonstrate the success of resolving what was initially concerning for a fixed delusion with patient-centered medication management and therapy. This case is presented as a vital contribution to the literature to bring awareness to a rare disorder with a poorly understood etiology that had a favorable outcome. Here, it is suggested that DMS may arise due to disrupted functional connectivity between highly coordinated brain networks, as evidenced by its occurrence in both organic neural disease and, as in this patient, trauma-related psychopathology.

## 1. Introduction

Delusional misidentification syndromes (DMSs) are a group of complex, monothematic delusional phenomena in which subjects hold a belief that the identity of a familiar person, object, location, or self has been altered or replaced. First described in the literature in 1923, Capgras Syndrome is characterized by the delusion that a close family member or friend has been replaced by an identical imposter. Reverse Capgras Syndrome is a DMS that refers to the self replaced by an imposter rather than a familiar other. Prior studies have found that most of the new identities patients took on were famous or admirable figures [1]. The majority of those patients also experienced a sudden awareness of the transformation or of the preexisting identity. Research on disorders with a likewise disturbance in reality testing and traumatogenic etiology, such as the dissociative subtype of posttraumatic stress disorder (PTSD), depersonalization-derealization disorder (DPDR), borderline personality disorder, and dissociative identity disorder (DID), has shed light on the neural processes involved in aberrations in self-referential processing. Here, we present the case of Ms. JS, a 25-year-old female admitted for first-episode psychosis and suicidal ideation in context of Reverse Capgras Syndrome.

## 2. Case Report

The patient was a 25-year-old female admitted for new-onset psychosis and suicidal ideation. She reported that she had not been herself for five days prior to admission and reported she did not believe her family recognized her. She also stated she was Jeffrey Epstein, and she endorsed this belief after seeing him appear on the television in the emergency room. She was tearful throughout the encounter and expressed a great deal of guilt, repeatedly stating that she should be in jail instead of the hospital. She reported paranoid delusions and auditory hallucinations of Epstein making derogatory remarks towards her. She also demonstrated some lapses in recent memory and explained that she had difficulty remembering some of her past life events.

Collateral from her mother revealed that this was the first time the patient exhibited delusions and paranoia. Her mother noted that the patient was recently in an abusive relationship where her partner had been controlling, threatening, and manipulative. The patient's mother also believed that the patient recently used marijuana the last time she was with her partner, which likely contributed in part to her psychosis. Brief psychotic disorder, schizoaffective disorder, major depressive disorder (MDD) with psychotic features, substance-induced psychosis, and complex PTSD were considered in the differential diagnoses. PTSD was unlikely due to a lack of overt intrusive, hyperarousal, and avoidance symptoms. While substance-induced psychosis was possible given the sudden onset of her psychotic features, it rarely lasts longer than a couple of days. The predominance of the patient's psychotic symptoms over depressive symptoms and duration of symptoms lasting less than one month in total made brief psychotic disorder the most appropriate diagnosis over MDD with psychotic features and schizoaffective disorder.

The patient was started on olanzapine 5 mg nightly. She attended group therapy every day, although she introduced herself as Jeffrey during group sessions for the first two weeks. She began to show gradual improvement in her distress tolerance, mood, memory of events prior to hospitalization, and reality testing. However, she continued to repeatedly express paranoid delusions, conviction that she should be in jail, auditory hallucinations of Epstein's voice, and occasional suicidal ideations in context of guilt and fear. Her dose of olanzapine was gradually titrated up, with careful monitoring of EKGs as she was tachycardic on most days, and QTc was intermittently prolonged at 485 and 472. She denied any cardiac symptoms throughout her hospitalization despite being tachycardic, and her QTc normalized on repeat EKG. She also continued to attend group therapy with progressively more active participation throughout her hospitalization. She was asked to rate how strongly she believed she was Jeffrey Epstein throughout her stay on a scale of 0 to 10, 0 being feeling like herself, and 10 meaning absolute conviction that she is Epstein. Until the second week of her stay, she rated her belief as 10 out of 10. During the time she rated her delusion as 10 out of 10, she was asked whether it was possible that she was not Epstein, to which she hesitantly answered “yes.” She rated her delusion as 5 out of 10 by her third week of hospitalization and stated that she believed she could eventually be 0 out of 10. Her olanzapine level was ultimately titrated up to 25 mg nightly, and her delusional rating steadily improved. Her serum olanzapine level was elevated at 114, but the patient remained on 25 mg nightly as she experienced no side effects, and her EKG showed no abnormalities.

During her fourth week of hospitalization, she rated her delusions as 0 out of 10. She shared at the end of her stay that she believed her psychotic episode was likely a trauma response from the abusive relationship she was recently in. She noted that she had low self-esteem and was easily influenced by the disparaging remarks her ex-partner made towards her. She reported that her low self-esteem evolved into ideas of reference, as she suspected other people were talking about her and that content she saw on television was targeted towards her. She was discharged after three and a half weeks of inpatient treatment feeling like her “true self,” not impersonating anybody. Her family also confirmed that they believe she has returned to her normal self and was happy to take her back home. Repeat olanzapine level obtained after discharge had normalized.

## 3. Discussion

DMSs such as Reverse Capgras Syndrome as exemplified by our patient JS have been explained from psychodynamic and neurobiological standpoints. One such explanation is that DMS may be a defense to detach from one's own unacceptable aspects and to project them onto an external figure [1]. Loss of familiarity, impaired self-monitoring, lack of ego boundaries, and attached emotional valence have also been noted as factors leading to such delusions [1]. In our case with JS, she was subject to frequent derogatory remarks from her abusive partner, leading to internalization of negative qualities. Instead of projecting those qualities onto an external figure, our patient introjected them and began to believe that she had been replaced by a famous figure, also of despicable qualities. Consistent with findings in the literature, she initially endorsed loss of familiarity with her mother whom she had always maintained a close relationship with, endorsed inability to distinguish herself from Epstein as she introduced herself as Jeffrey during group sessions while also responding to her name when called upon, and endorsed similar feelings of hatred and disgust towards Epstein as she did for herself when she was abused by her partner.

Emerging literature points to evidence for links between dissociative states and altered activity across brain networks responsible for the processing of emotions, memory, attention, and interoception—all of which contribute a role in the perception of self-understanding. One of the earliest models proposed to explain dissociation as a maladaptive response to trauma is the theory of “corticolimbic disconnection.” Overstimulation of regions involved in emotion, salience detection, and stress responses (i.e., the anterior cingulate cortex and the amygdala) modulate ascending arousal systems which activate the nondominant prefrontal cortex (PFC). Activation of frontal regions leads to a reciprocal inhibitory element mediated by the PFC that impedes limbic structures involved in emotional experiencing and dampens sympathetic outputs [2]. As a result, a traumatized individual may experience emotional numbing, subjective detachment, and feelings of emptiness. While limbic activity is markedly dampened, increased PFC activation leads to symptoms associated with imbalanced cognitive control and attention. This may manifest in excessive rumination, hypervigilance, and errors in threat appraisal.

Capgras Syndrome has long been associated with neurological diseases, especially damage to the nondominant hemisphere. The sequelae of these kinds of lesions entail not only the belief a close family member is an impostor, but more broadly, a significant affective dissonance [3]. Common between all delusional misidentification syndromes is the stark loss of familiarity with self, others, or the environment. As the amygdala is highly implicated in emotional responses to external stimuli as well as the storage of implicit procedural memories, a hypoactive limbic system would indicate difficulty recalling firmly etched past experiences. Whereas the hippocampus primarily stores verbal, declarative memories, the amygdala stores their personal salience, or “affective quality.” If memories reach a certain traumatic threshold, the hippocampus may not store an explicit memory trace, as seen in dissociative amnesia, because it cannot effectively contextualize the threat. Indeed, it is established that hippocampal volume shrinks in cases of PTSD and chronic stress [2].

Feelings of alienation from one's environment may be conceptualized the same way as derealization, and unfamiliarity with oneself as depersonalization, the two main symptomatic components of dissociation across different trauma-related stress disorders. However, feelings of detached strangeness may still primarily be confined to a specific modality. For instance, the modality of vision in Capgras Syndrome when viewing significant others' faces supports a view of visuolimbic disconnection. Neuroimaging studies investigating DID and DPDR have observed abnormal face processing in these patients [2]. A corollary to this is the inability of JS to recognize herself in both a mirror and in her own mind.

Dissociative symptoms alone however do not completely account for a fixed, monothematic delusion. While DMS such as Reverse Capgras may involve a severe form of depersonalization, it is also a failure to match current experience to preexisting emotional information. In the case of JS, confabulated imaginary differences were used to reconcile incongruent beliefs about herself. This may in part be due to executive functions that normally correct errors of self-judgment becoming impaired in the setting of severe frontolimbic disconnection. Working memory is one of the most crucial executive functions for one's self-view, involving ongoing integration of stored long-term memories with newly acquired information from the environment. Destabilization of these neuronal connections may render a patient incapable of reevaluating their delusion; the “working self” that experiences life via interaction with its surroundings and the “longitudinal self” of preencoded memories are no longer organized as one entity [4]. In other disorders of serious identity disturbance such as DID, trauma throughout childhood development disturbs the structure of self-continuity over time; episodic memories which require storage, encoding, and retrieval of emotional salience are interrupted in exchange for an attentional pattern designed to adapt to chronic threats to survival.

Though cases of DMS are rare, the literature reveals these syndromes often intersect and transform into each other. Patients tend to pass through a continuum of increasing self-disorganization and may cumulate into syndromes like Cotard's delusion involving a complete deterioration of identity expressed as psychological death [4]. However, our patient JS exemplifies a case of DMS that progressed to complete resolution rather than transforming or deteriorating into other dismal outcomes. Her disturbances in sense of self, executive functioning, affective memory, and facial processing/recognition were temporary, which further supports the contribution to the literature that although complex, DMS can be seen to have favorable outcomes.

## 4. Conclusion

Given the rarity of DMS, there are currently no specific treatments for Reverse Capgras Syndrome. Though DMS often overlap and appear to share similar psychodynamic and neurobiological correlates, classifying their phenomenological distinctions is useful in determining the best therapeutic approach for each patient. Most other cases of DMS have been treated with experimental psychopharmacology focusing on coexisting psychotic disorders, usually atypical antipsychotics or technology such as transcranial magnetic stimulation, neurofeedback, or transcranial direct current stimulation [5]. However, many cases of DMS symptoms remain resistant to treatment. In our case, JS's concomitant brief psychotic disorder was targeted with olanzapine and therapy leading to successful resolution of her symptoms. Our case highlights the importance of delving deeper into one's psychosocial history, particularly their trauma history, to grasp a fuller understanding of such rare cases. In doing so, clinicians may advocate for incorporating therapy alongside medications not only for mood and anxiety disorders in the traditional outpatient setting but also for acute psychotic conditions in the inpatient unit for a more optimistic prognosis than many prior cases of DMSs.
